# The value of combined application of ultrasound-guided fine needle aspiration cytology and thyroglobulin measurement for the diagnosis of cervical lymph node metastases from thyroid cancer

**DOI:** 10.12669/pjms.315.6726

**Published:** 2015

**Authors:** Jia-hong Shi, Ying-ying Xu, Qi-zheng Pan, Guo-qing Sui, Jian-ping Zhou, Hui Wang

**Affiliations:** 1 Jia-hong Shi, Department of Ultrasonography, China-Japan Union Hospital of Jilin University, Changchun 130033, China; 2 Ying-ying Xu, Department of Ultrasonography, China-Japan Union Hospital of Jilin University, Changchun 130033, China; 3 Qi-zheng Pan, Department of Anesthesia, China-Japan Union Hospital of Jilin University, Changchun 130033, China; 4 Guo-qing Sui, Department of Ultrasonography, China-Japan Union Hospital of Jilin University, Changchun 130033, China; 5 Jian-ping Zhou, Department of Ultrasonography, China-Japan Union Hospital of Jilin University, Changchun 130033, China; 6 Hui Wang, Department of Ultrasonography, China-Japan Union Hospital of Jilin University, Changchun 130033, China

**Keywords:** Cervical lymph node, Fine needle aspiration cytology, Papillary thyroid cancer, Thyroglobulin

## Abstract

**Objective:**

The aim of this study was to explore the diagnostic value of ultrasound-guided (US-guided) fine-needle aspiration cytology (FNAC), thyroglobulin measurement on fine-needle aspiration (FNA-Tg), combined US-guided FNAC, and the ratio between FNA-Tg and serum Tg (FNA-Tg/serum Tg) for patients with cervical lymph node (CLN) metastases from thyroid carcinoma.

**Methods:**

We selected 148 patients with thyroid cancer with suspicious CLN metastases who met the inclusion criteria. FNAC findings, FNA-Tg levels, and serum Tg levels were evaluated before surgical treatment. The results of FNAC and FNA-Tg from CLNs were analyzed retrospectively.

**Results:**

Ninety-four of 148 cases were metastatic and 54 were benign. The sensitivity, specificity, and accuracy of FNAC were 68.1%, 100.0%, and 79.7%, respectively. The sensitivity, specificity, and accuracy of FNA-Tg/serum Tg were 91.5%, 88.9%, and 90.5%, respectively. The sensitivity, specificity, and accuracy of FNA-Tg [10 ng/mL] were 98.9%, 68.5%, and 87.8%, respectively. The sensitivity, specificity, and accuracy of combined US-guided FNAC and FNA-Tg/serum Tg were 95.7%, 96.3%, and 95.9%, respectively. There was a statistically significant difference between FNAC and combined US-guided FNAC and FNA-Tg/serum Tg for sensitivity, specificity, and accuracy (P < 0.05).

**Conclusion:**

The method of FNA-Tg/serum Tg is sensitive enough for diagnosing CLN metastases from thyroid cancer. The combined application of US-guided FNAC and FNA-Tg/serum Tg contributes to improving the accuracy of diagnosing CLN metastases in patients with thyroid cancer.

## INTRODUCTION

After the development of diagnostic technology (ultrasonography (US) and fine-needle aspiration cytology (FNAC)), thyroid cancer became the most common endocrine tumor and its incidence increases each year.^1^-^4^ Although the prognosis of thyroid cancer is often good after initial surgical treatment, the focal or regional recurrence rate is 5%-20% and the probability of a distant metastasis is 10%-15%.^5^-^7^ Therefore, early detection of cervical lymph node (CLN) metastases from thyroid cancer is particularly important for guiding clinical treatment and the selection of surgical methods.

In recent years, two diagnostic methods (US-guided FNAC and detection of thyroglobulin on FNA (FNA-Tg)) are often used to diagnose suspicious CLN metastases from thyroid cancer. US-guided FNAC can be a relatively trustworthy method to detect suspicious CLNs but it has the limitation of a 6%-8% false-negative rate.^7^,^8^ The application of FNA-Tg can be a supplemental and excellent technique to detect CLN metastases in patients because it cannot be affected by the level of Tg antibodies (TgAb) in serum.^4^,^9^ However, there is no appropriate cut-off value of FNA-Tg, and it can range between 0.2 ng/ml and 100 ng/ml.^9^ The two methods mentioned above have their own clinical advantages and disadvantages that can compensate for each other when used in conjunction. FNAC has a relatively high sensitivity and accuracy for diagnosis of CLN metastases, but it is limited in that its results depend on the pathologists’ experience and the quantity of cells recovered. FNA-Tg has the advantage of having a high sensitivity for cystic components in lymph nodes.^6^

Therefore, it seems that combining US-guided FNAC with FNA-Tg can improve the accuracy of early diagnosis of CLN metastases from thyroid cancer. However, to the best of our knowledge, the diagnostic performance of combined US-guided FNAC and FNA-Tg/serum Tg has not been tested before. Therefore, this study was conducted to estimate the diagnostic value of US-guided FNAC, FNA-Tg, and the combined application of US-guided FNAC and FNA-Tg/serum Tg for detecting CLN metastases from thyroid cancer.

## METHODS

### Patients

We selected 148 patients with thyroid cancer with suspicious CLN metastases treated from August 2012 to December 2013 at China-Japan Union Hospital of Jilin University in Changchun. FNAC findings, FNA-Tg levels, and serum Tg levels were evaluated before the operation. The suspicious CLNs that were located before lymphadenectomy were removed and then examined by histopathology.

This study was conducted in accordance with the declaration of Helsinki and with approval from the Ethics Committee of Jilin University. Written informed consent was obtained from all participants.

The patients who participated in our study had to meet the following inclusion criteria: [1] diagnosis of papillary thyroid cancer (PTC) confirmed on pathology after initial thyroidectomy and lymph node excision; [2] pre-surgical examinations including ultrasonography, FNAC, FNA-Tg, and serum Tg had to show the presence of suspicious CLNs; [3] suspicious CLNs had to be round, without clear cortex and medulla, an aspect ratio of <1.5, microcalcifications, a rich blood supply, and the type of PW (high speed and low resistance).

Exclusion criteria of our study were [1] pathologic diagnosis including thyroid diseases other than PTC, [2] a history of cervical surgery or distant metastases of PTC, [3] the presence of other cervical neoplasms, [4] and other serious diseases such as heart diseases or nervous system problems.

## RESULTS

### Pathology

All 148 cases were confirmed as PTC by primary pathology. Ninety-four were metastatic and 54 were benign.

### FNAC

Sixty-four of 94 metastatic CLNs were positive and 30 cases were negative. All 54 benign CLNs were negative on FNAC. The sensitivity, specificity, and accuracy of FNAC were 68.1%, 100.0%, and 79.7%, respectively.

### FNA-Tg

When using 1 as the cut-off value for FNA-Tg/serum Tg, 86 of 94 metastatic CLNs were positive and eight cases were negative. Forty-eight of 54 benign CLNs were negative and six were positive. The sensitivity, specificity, and accuracy of FNA-Tg/serum Tg were 91.5%, 88.9%, and 90.5%, respectively.

When using 10 ng/mL as the cut-off value for the FNA-Tg measurement, 93 of 94 metastatic CLNs were positive and one case was negative. Thirty-seven of 54 benign CLNs were negative and 17 were positive. The sensitivity, specificity, and accuracy of FNA-Tg [10 ng/mL] were 98.9%, 68.5%, and 87.8%, respectively.

### Combined

Of the 148 cases, 90 of 94 metastatic CLNs were positive and four cases were negative. Fifty-two of 54 benign CLNs were negative and two were positive. The sensitivity, specificity, and accuracy of the combined test were 95.7%, 96.3%, and 95.9%, respectively. The results are summarized in Table I.

**Table-I:** The results of FNAC, FNA-Tg and the combined US-guided FNAC and FNA-Tg/serumTg about 148 lymph nodes

Method	Examination/Pathology				Sensitivity (%)	Specificity (%)	Positive predictive value (%)	Negative predictive value (%)	Accuracy (%)
	+/+	+/-	-/+	-/-					
FNAC	64	0	30	54	68.1	100	100	64.3	79.7
FNA-Tg/serumTg	86	6	8	48	91.5	88.9	93.5	85.7	90.5
FNA-Tg[10ng/ml]	93	17	1	37	98.9	68.5	84.5	97.4	87.8
FNAC+FNA-Tg/serumTg	90	2	4	52	95.7	96.3	97.8	92.8	95.9

+, Positive; -, Negative.

### Statistical analysis

There was no statistically significant difference between FNA-Tg/serum Tg and FNA-Tg [10 ng/mL] (P > 0.05).

The sensitivity of FNA-Tg/serum Tg was significantly higher than that of FNAC. The differences were statistically significant (P < 0.05), but there was no statistically significant difference between their specificities.

There was a statistically significant difference for sensitivity, specificity, and accuracy between FNAC and the combined application of US-guided FNAC and FNA-Tg/serum Tg (P < 0.05).

## DISCUSSION

Positron emission tomography (PET)/computed tomography (CT) is more sensitive than other imaging methods including ^131^I, CT, magnetic resonance imaging (MRI), and ultrasonography for the detection of CLN metastases from thyroid cancer.^10^-^12^ However, it is not widely used because of its high cost and unavailability. On the other hand, US-guided FNAC is a relatively economical and common technique to diagnose CLN metastases in patients with thyroid cancer.^7^,^8^,^13^

Among the 148 cases in our study, there were 30 false-negatives on FNAC. Seventeen were the result of liquefied lymph node suction that had no exact cellular structure. The remaining thirteen cases might be due to any of a number of reasons, such as the selection of the lymph node, the puncture technique, the smear, a deficiency of cell volume, or the skills of the pathologists. In order to improve the accuracy of FNAC, it is helpful to conduct multiple lymph node punctures and consult multiple pathologists.

In our study, the sensitivity, specificity, and accuracy of FNAC were 68.1%, 100.0%, and 68.1%, respectively. Giovanella et al.^7^ reported the sensitivity, specificity, and accuracy of FNAC as 71%, 80%, and 74%, respectively. Their results are in agreement with ours.

Thyroid tissue synthesizes and secretes Tg. Tg in blood comes from thyroid tissue, and its concentration in the blood depends on the size of the thyroid, the degree of damage, hormones, and TgAb. In general, the size of thyroid is the main factor affecting the level of serum Tg, and the secretion of thyroid stimulating hormone (TSH) from the pituitary is positively correlated with the serum Tg level.

Most domestic clinicians advocate total or near-total thyroidectomy with or without lymph node dissection as the initial treatment for patients with thyroid cancer.^14^ In recent years, the domestic application of FNA-Tg for diagnosing CLN metastases of thyroid cancer has attracted more attention. The lymph node eluent of Tg has no correlation with TgAb and TSH. This means that even when tests for TgAb in the serum are positive, FNA-Tg measurements are still reliable. Therefore, FNA-Tg has an advantage for detecting CLN metastases.^9^,^15^

In our study, we chose two cut-off values: 1 for FNA-Tg/serum Tg and 10 ng/ml for FNA-Tg. There was no statistically significant difference between the two values for sensitivity, specificity, or accuracy (P < 0.05). The specificity and accuracy of FNA-Tg/serum Tg is better than that of FNA-Tg [10 ng/ml]. Thus, we should select the method of FNA-Tg based on the sensitivity, specificity, positive predictive value, negative predictive value, and accuracy. Our results suggest that using 10 ng/ml as the cut-off has such a high false-positive rate that its application might be limited.

Borel et al.^16^ reported two cases of lymph node metastasis confirmed by cytology that had FNA-Tg values of only 7.1 ng/ml and 6.6 ng/ml. In our study, the FNA-Tg value of one case was 7.9 ng/ml. Thus, some scholars suggest the use of 8.0 ng/ml as the cut-off value.^17^ Giovanella et al.^7^ suggested that the sensitivity, specificity, positive predictive value, negative predictive value, and accuracy of 1.1 ng/ml were all 100.0% through the comparative analysis of 126 patients. The diverse opinions above show that there is no unified standard for the cut-off value of FNA-Tg.

The ratio of FNA-Tg/serum Tg reduces errors by excluding any effect of Tg in serum. The specificity and positive predictive value of the ratio can compensate for the deficiency of FNA-Tg. We believe that FNA-Tg/serum Tg can be an important method for diagnosing CLN metastases from thyroid cancer.

FNAC has become the primary method for diagnosing CLN metastases, with a sensitivity and accuracy, but it still depends directly on the pathologists’ experience and quantity of cells aspirated. In order to solve these problems, FNA-Tg can be a favorable technique because of its sensitivity for cystic components in lymph nodes.^6^

As an accurate method to detect CLN metastases, FNA-Tg is better than FNAC for accuracy in detecting cystic lymph nodes.^18^ In our work, there were eight false-negative cases by FNA-Tg/serum Tg, which were caused by puncture deviation and suction of the adipose tissue. In addition, there were six false-positive cases. The reason for these cases might be complicated in that Tg in the blood was influenced by TSH and TgAb levels. This resulted in lower levels of detected Tg.

It is obvious that the combined application of US-guided FNAC and FNA-Tg/serum Tg has higher sensitivity and accuracy than FNAC and higher sensitivity, specificity, and accuracy than FNA-Tg/serum Tg. Cunha et al^6^ reported results that are in accordance with ours.

In conclusion, combined US-guided FNAC and FNA-Tg/serum Tg can improve the accuracy of diagnosing CLN metastases in patients with thyroid cancer by compensating for the deficiencies of each technique.
